# An unusual cause of pacemaker malfunction: A case report of an association of twiddler and reel syndrome

**DOI:** 10.1016/j.amsu.2022.104857

**Published:** 2022-11-11

**Authors:** Achraf Machraa, Oussama Sidaty, Nadia Fellat, Rokaya Fellat

**Affiliations:** Dept. of Cardiology A, National Cardiovascular League, Ibn Sina University Hospital Center, Mohammed V University, Rabat, Morocco

**Keywords:** Lead dislodgment, Coiling lead, Twiddler syndrome, Reel syndrome, Case report

## Abstract

**Introduction and importance:**

Lead dislodgement syndromes (Twiddler, Ratchet or Reel syndromes) are rare causes of cardiac stimulation device malfunction that can occur most commonly early after device implantation. Each one of them associated with a unique pattern of lead coiling and dysfunction. Our clinical case reports an unusual association and shed the light on the available diagnostic modalities.

**Case presentation:**

A 62-year-old woman who was referred to our hospital for a symptomatic high degree AV block, she underwent dual chamber pacemaker implantation. She experienced 3 weeks following implantation a rhythmic twitching of the right arm without syncope. The device interrogation revealed an increase in both leads pacing impedance and chest X-ray showed leads had pulled out of the heart and were tangling and wrapped repeatedly around the pulse generator. Revision procedure was performed to reposition the leads.

**Clinical discussion:**

Recognizing this complication early can prevent life threatening complication and is then of the utmost importance. Twiddler's syndrome is due to rotation of the device along its long axis. Reel syndrome is produced by device rotation along the transverse axis. In most cases, lead replacement or reposition is needed. Preventive measures such as patient education and use of a smaller pocket will reduce the risk of developing the syndrome.

**Conclusion:**

Our case highlights the available diagnostic modalities for early detection of twiddler's syndrome. The unique nature of this case increases the importance of considering device lead dislodgement as the cause for patients presenting with extra-cardiac symptoms.

## Highlights

• Patients with cardiac implantable devices who present with neuromuscular symptoms should be suspected to have a device lead dislodgement.
• The patient should undergo an ECG, chest X-ray and cardiac monitoring until a device interrogation can be performed.
• Appropriate implantation technique is one of the solutions for preventing lead dislodgment syndromes.
• Surgical pocket revision should be combined with lead screening for any signs of insulation leakage or conduction damage.

## 1 Introduction

First described by Bayliss et al., in 1968. The term «twiddler syndrome» is used to describe the dysfunction of a cardiac implantable electronic devices (CIED) lead due to direct manipulation or spontaneous rotation of the generator [1]. It is an unusual condition in which a patient's subconscious, unintended, or intentional external manipulation of their cardiac device results in the movement of the transducer wires. At the present time, three different device rotation syndromes have been described (twiddler, reel and ratchet), each one of them associated with a unique pattern of lead coiling and dysfunction. Surgical pocket-revision with lead reposition or replacement is needed in most cases. In this case report, we describe a patient with a dual chamber pacemaker who presented with rhythmic arm twitching after 3 weeks of implantation and was afterward diagnosed as having an association of twiddler and reel syndrome.

This case report has been reported in line with the SCARE Criteria [2].

## 2 Timeline

**Table:** 

Three weeks prior to admission	The patient, presenting with lipothymic discomfort, dyspnea and fatigue, underwent a dual-chamber pacemaker implantation due to a 2:1 atrioventricular block, with normal pacing, sensing and threshold parameters.
Admission	The patient described a rhythmic twitching of the right arm, the electrocardiography showed a 2:1 atrioventricular block. Check-up of the device revealed an abrupt increase in both atrial and ventricular pacing impedance. A chest X-ray showed a dislodgement of the right atrial and ventricular lead. Both leads were tangling and wrapped around the pacemaker generator.
Day 2	We performed a second operation, both leads were successfully repositioned. The day after, the patient was discharged
Four weeks after admission	Check-up of the device showed normal pacing, sensing, and threshold parameters. The patient experienced no further recurrence of symptoms at follow-up.

## 3 Case presentation

A 62-year-old woman (body height 150 cm, body weight 87 kg) with a past medical history of hypertension, well controlled by Amlodipine 5mg, was referred to our hospital because of severe bradycardia. The presenting symptoms were lipothymic discomfort, shortness of breath and fatigue evolving for 2 weeks. The resting electrocardiogram (ECG) demonstrated 2:1 atrioventricular block. A dual chamber pacemaker was implanted via the right cephalic vein. A screw-in ventricular lead was placed at the right ventricular and another screw-in atrial lead was placed at the right atrial appendage. Fixation of both leads was done in the standard fashion by suturing on lead sleeve with silk suture. The pacemaker generator was then connected to both leads and placed into a subcutaneous tissue pocket. The procedure was carried out uneventfully. Her after surgery ECG confirmed that pacemaker function was good. Prior to discharge a chest X-ray was taken and also confirmed that the pacemaker generator and its leads were in proper position.

**Fig. 1:**
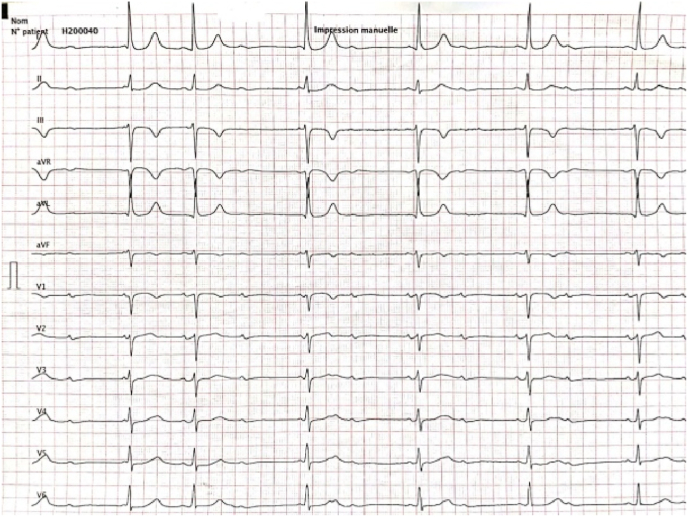
ECG showing her own rhythm back to 2:1 atrioventricular block.

**Fig. 2:**
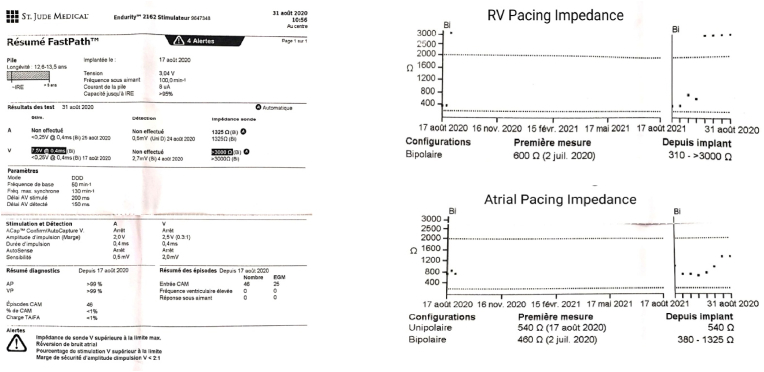
Device trends obtained at interrogation indicate a large increase in the pacing impedance values in both leads.

**Fig. 3:**
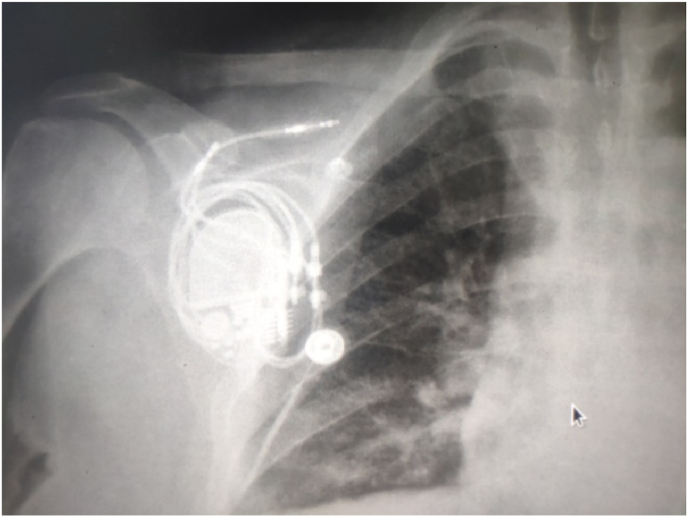
Dislodgement of the right atrial and ventricular lead. Both leads were tangling and wrapped around the pacemaker generator.

Three weeks after the procedure, the patient was reported to have experienced a rhythmic twitching of the right arm for the past week without syncope or lipothymic discomfort. The clinical examination did not reveal any abnormality. Laboratory tests were unremarkable. The ECG showed her own rhythm back to 2:1 AV block (Fig. 1). She was then referred again to our hospital for further investigation. The pacemaker interrogation revealed an abrupt increase in both atrial and ventricular pacing impedance (up to 1325Ω in the atrial lead, and up to 3000Ω in the ventricular lead). The trends as reported by the device interrogation are shown in Fig. 2. A chest X-ray was urgently performed and showed that the pacing leads were tangling and wrapped repeatedly around the pulse generator (Fig. 3).

The diagnosis of lead dislodgment with an association of Twiddler and Reel syndrome was made. The main differential diagnosis for patients who may present with rhythmic neuromuscular contractions includes tremor, myoclonus, tic disorder, chorea, asterixis …

It was discovered that the patient kept rubbing and scratching her shoulder near her pacemaker pocket site due to eczema, probably secondary to a povidone iodine (betadine) allergy, with a spontaneous resolution, but she denied manipulating the device itself.

**Fig. 4:**
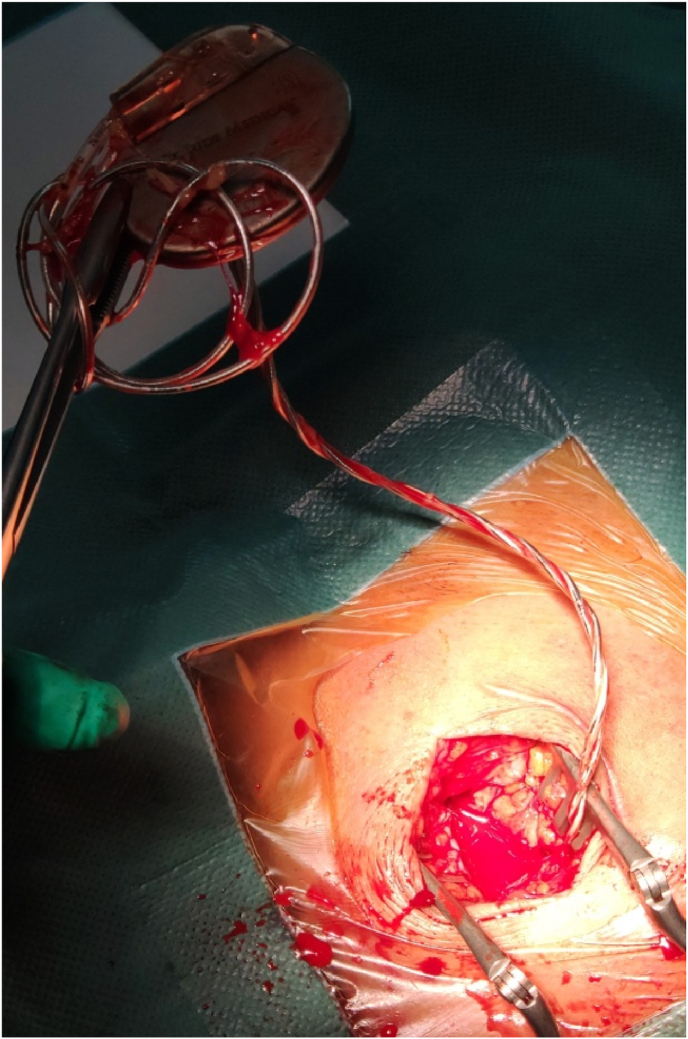
Peroperative picture confirm the findings of the chest X-ray, Both leads were found tangling and wrapped around the pacemaker generator without any signs of structural damage.

We performed a second operation to reposition the leads. The patient received antibiotic prophylaxis before the revision procedure. The leads were found twisted and wrapping the pacemaker generator without any signs of insulation leakage and conduction damage (Fig. 4). Both pacemaker leads were successfully repositioned, into the right ventricular apex and the right atrial appendage, and meticulously fixed with nonabsorbable suture on its sleeve with surrounding fascia and muscles. Additional sutures were added to secure the pacemaker generator to the pectoral muscle in the surgical pocket.

**Fig. 5:**
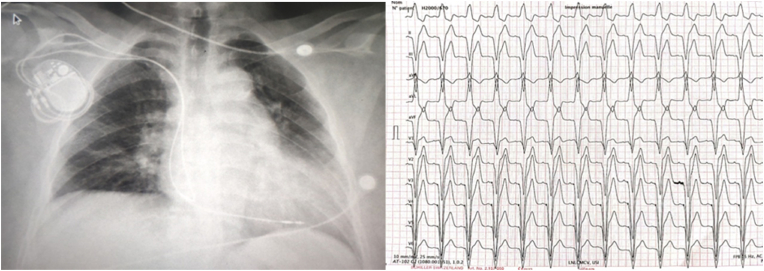
**A.** Figure shows device on the right side and leads in the appropriate position. **B.** Figure shows a dual-chamber pacing ECG.

A chest X-ray (Fig. 5A) and ECG (Fig. 5B) were obtained after the procedure to ensure the absence of pneumothorax and to document the appropriate functioning of the device. The patient was discharged the following day, risk mitigating techniques were provided: Avoiding device manipulation, avoiding excessive shoulder rotation and abduction, wearing a broad arm sling.

After subsequent follow up, the patient was well, the pacemaker function was good, with normal pacing, sensing and threshold parameters, and no complications have been noticed.

## 4 Discussion

We report a case presentation of an elderly woman with both Twiddler and Reel syndrome, presenting with symptoms suggestive of neuromuscular stimulation.

Lead dislodgement syndromes (Twiddler, Ratchet or Reel syndromes) are rare causes of cardiac stimulation device malfunction that can occur most commonly early after device implantation.

**Fig. 6:**
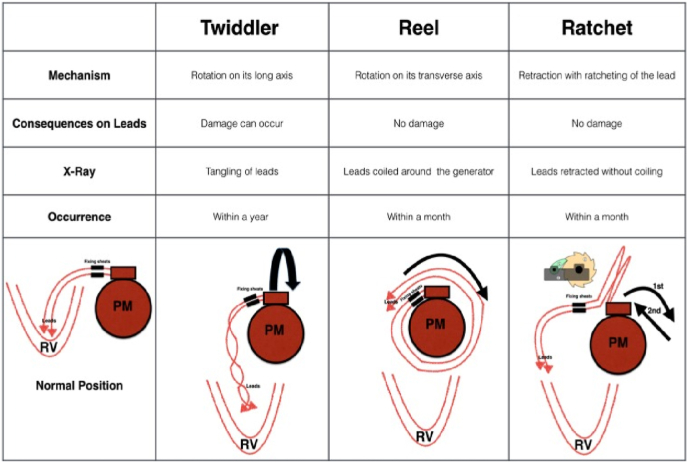
Differences among the dislocation lead-dysfunctioning syndromes.

They differ from each other in the causing mechanism (Fig. 6). Twiddler's syndrome is due to rotation of the generator on its long axis, causing twisting of the leads. On the other hand, Reel syndrome is due to rotation of the device on its transverse axis leading to the coiling of the leads around the generator without any damage to the leads. Ratchet syndrome involves upward and downward shoulder motion causing progressive retraction of the leads without twisting or coiling around the device [3,4].

Most cases of reel syndrome occur within a month of implantation, whereas twiddler's syndrome can occur later, up to one year from implantation. In our case, this complication occurred within 3 weeks after implantation. The most common risk factors for twiddler's syndrome are elderly patients, female, and large device pocket [5]. Other risk factors are sports which include wide and repetitive movements of the arms, such as golfing and swimming.

This phenomena causes wide array of symptoms, most notably dysrhythmias, syncope or near syncope, exacerbation of heart failure. Although cardiac symptomatology predominates, the stimulation of the phrenic nerve by the dislodged leads can cause diaphragmatic contractions and the stimulation of the brachial plexus can cause rhythmic arm twitching [6], which was the case for our patient. Diagnosis is easily made by chest X-ray, which normally show dislodgement of the lead, ECG and device interrogation are also performed routinely for diagnostic purposes [7].

Preventive measures such as patient education and use of a smaller pocket will reduce the risk of developing the syndrome. Suturing the pacemaker generator into surrounding tissue, as well as placing the generator into the pectoral muscle may also prevent the syndrome. Other preventive measures include using a compression band around the upper chest and shoulder and tightening of the patient's arm for at least five to seven days [8].

The unique nature of this case increases the importance of considering device lead dislodgement as the cause for patients presenting with extra-cardiac symptoms. Pacemaker lead stimulation of surrounding structures can present in an unusual fashion, masking the diagnosis. Lead dislodgement should always be considered as a differential diagnosis in patients with pacemaker presenting with neuromuscular symptoms since rapid recognition and intervention are critical in preventing potential life threatening complications.

## 5 Conclusion

Our case highlights the available diagnostic modalities for early detection of twiddler's syndrome. The unique nature of this case increases the importance of considering device lead dislodgement as the cause for patients presenting with extra-cardiac symptoms.

## Ethical approval

Not applicable.

## Sources of funding for your research

The authors declare that this work was not supported by any grants or funding support

## Author contribution

Achraf Machraa was involved in the study concept, the collection of the data, drafting, literature review, and editing of the manuscript.

Oussama Sidaty was responsible for literature review and revising the manuscript for important intellectuel content.

Nadia Fellat was responsible of data validation and supervision.

Rokaya Fellat was responsible of data validation and supervision.

## Registration of research studies

This is not and original research project involving human participants in an interventional or an observational study but a case report, this registration was not required.

## Consent

Written informed consent was obtained from the patient for publication of this case report and accompanying images. A copy of the written consent is available for review by the Editor-in-Chief of this journal on request.

## Guarantor

Achraf Machraa.

## Availability of data and materials

The data is available for sharing.

## Provenance and peer review

Not commissioned, externally peer-reviewed.

## Declaration of competing interest

All the authors declare that they have no competing interests.
